# Animal models of *Klebsiella pneumoniae* mucosal infections

**DOI:** 10.3389/fmicb.2024.1367422

**Published:** 2024-03-15

**Authors:** Lucas Assoni, Ana Julia Melo Couto, Brenda Vieira, Bárbara Milani, Alice Souza Lima, Thiago Rojas Converso, Michelle Darrieux

**Affiliations:** Laboratório de Microbiologia Molecular e Clínica, Universidade São Francisco, Bragança Paulista, Brazil

**Keywords:** *Klebsiella pneumoniae*, animal models, disease pathogenesis, mucosal infection, pre-clinical

## Abstract

*Klebsiella pneumoniae* is among the most relevant pathogens worldwide, causing high morbidity and mortality, which is worsened by the increasing rates of antibiotic resistance. It is a constituent of the host microbiota of different mucosa, that can invade and cause infections in many different sites. The development of new treatments and prophylaxis against this pathogen rely on animal models to identify potential targets and evaluate the efficacy and possible side effects of therapeutic agents or vaccines. However, the validity of data generated is highly dependable on choosing models that can adequately reproduce the hallmarks of human diseases. The present review summarizes the current knowledge on animal models used to investigate *K. pneumoniae* infections, with a focus on mucosal sites. The advantages and limitations of each model are discussed and compared; the applications, extrapolations to human subjects and future modifications that can improve the current techniques are also presented. While mice are the most widely used species in *K. pneumoniae* animal studies, they present limitations such as the natural resistance to the pathogen and difficulties in reproducing the main steps of human mucosal infections. Other models, such as *Drosophila melanogaster* (fruit fly), *Caenorhabditis elegans, Galleria mellonella* and *Danio rerio* (zebrafish), contribute to understanding specific aspects of the infection process, such as bacterial lethality and colonization and innate immune system response, however, they but do not present the immunological complexity of mammals. In conclusion, the choice of the animal model of *K. pneumoniae* infection will depend mainly on the questions being addressed by the study, while a better understanding of the interplay between bacterial virulence factors and animal host responses will provide a deeper comprehension of the disease process and aid in the development of effective preventive/therapeutic strategies.

## Introduction

*Klebsiella pneumoniae* is a widely distributed bacterium that colonizes the human skin, mouth, respiratory and gastrointestinal (GI) tracts asymptomatically. It is also one of the main causative agents of hospital infections such as urinary tract infections, pneumonia, liver abscesses, meningitis, and sepsis, being considered an opportunistic pathogen (Conlan et al., 2012; Gorrie et al., 2017; Joseph et al., 2021; Osbelt et al., 2021). In addition, *K. pneumoniae* is among the most relevant strains when considering the increase in antibiotic resistance worldwide, being classified by the World Health Organization (WHO) as a priority pathogen for which new drugs are needed (WHO, 2017). However, relying on the discovery of new drugs alone may not be enough to suppress the advance of these infections, especially when caused by multidrug-resistant isolates, so new strategies are fundamental to stop the steady increase of cases.

Infections caused by multi-drug resistant bacteria (including *K. pneumoniae*) pose a great burden to healthcare systems worldwide, from the hundreds of thousands of deaths, to the reduced life expectancy and disabilities, along with the cost of the treatment. *K. pneumoniae* is among the four main causes of death by antibiotic resistance bacteria, which are responsible for 929,000 annual deaths according to a study published in 2022 (Antimicrobial Resistance Collaborators, 2022).

*K. pneumoniae* strains can be classified in two major categories, namely classical (or common) and hypervirulent strains, based on traits such as the hypermucoviscosity (HMV) phenotype and increased expression of siderophores and fimbriae (Chang et al., 2021; Dai and Hu, 2022). There are a few differences in the profile of the infections caused by those types of *K. pneumoniae*. Usually, most cases occur within healthcare environments and are caused by classical strains. Such strains are commonly multi-drug resistant, especially to beta-lactams, including carbapenems. The most common infections caused by the classical strains are urinary tract infections, pneumonia and bacteremia (Paczosa and Mecsas, 2016; Dai and Hu, 2022).

**Figure 1:**
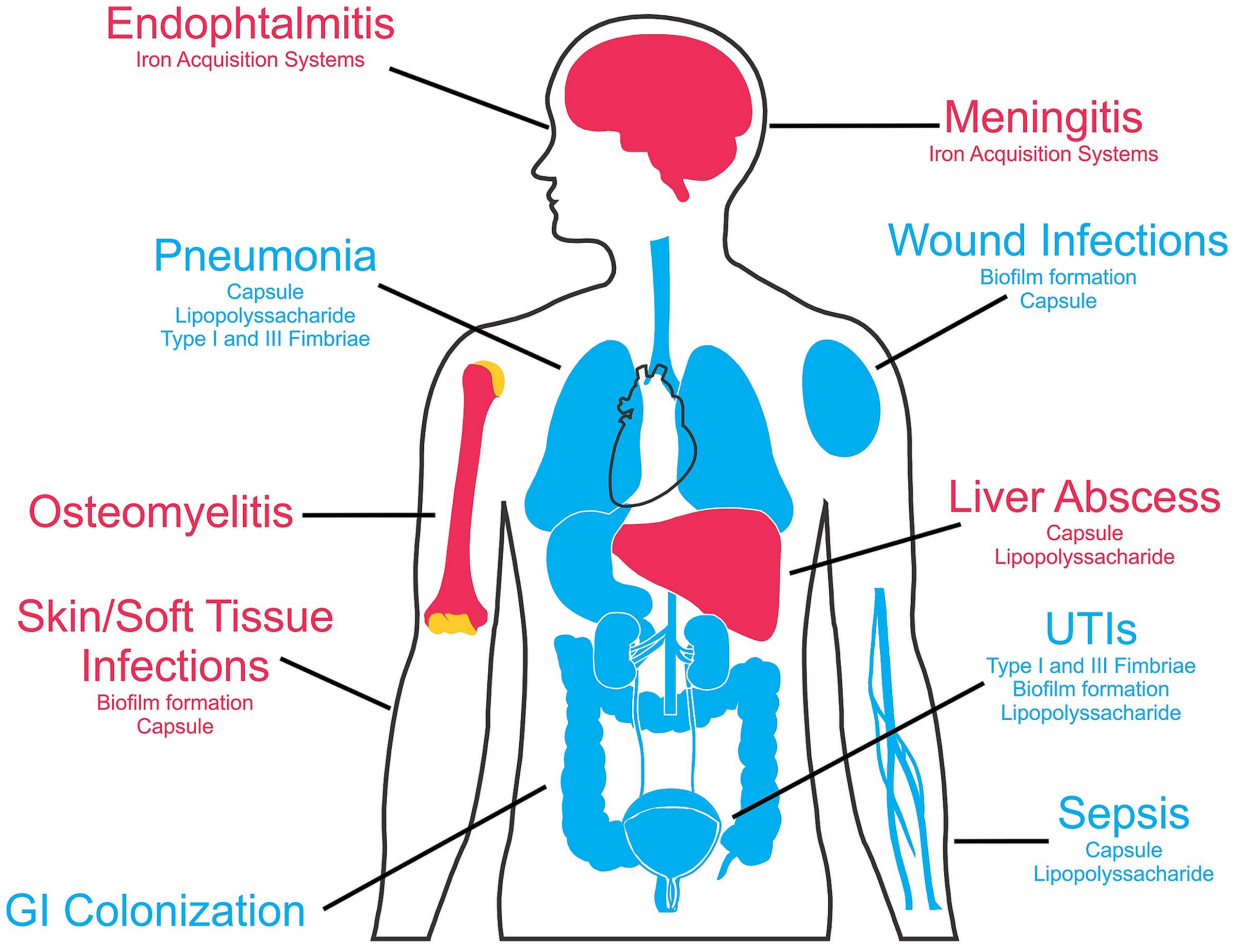
*Klebsiella pneumoniae* human infection sites and main associated virulence factors. The blue color indicates the infections caused by classical *K. pneumoniae* strains, while diseases commonly associated with hypervirulent strains are marked in red. This figure was partly generated using templates from Servier Medical Art (Servier), and SlidesGo (Freepik), licensed under a Creative Commons Attribution 3.0 unported license.

Hypervirulent strains, on the other hand, are acquired in the community and are more invasive, being able to colonize additional sites and cause further damage, when compared to the classical strains. Another important difference between classical and hypervirulent strains is the historically higher susceptibility of the hypervirulent strains to antimicrobials, which is becoming less prevalent in hospital-acquired *K. pneumoniae* in many regions, especially in lower- or middle-income countries (Dai and Hu, 2022; Pulingam et al., 2022). Nevertheless, a very worrying development is the increase in reports of resistant hypervirulent strains leading to more severe, often fatal infections (Choby et al., 2020; Dai and Hu, 2022). A compilation of the most common infections caused by classical and hypervirulent strains of *K. pneumoniae* is shown in Figure 1.

**Figure 2:**
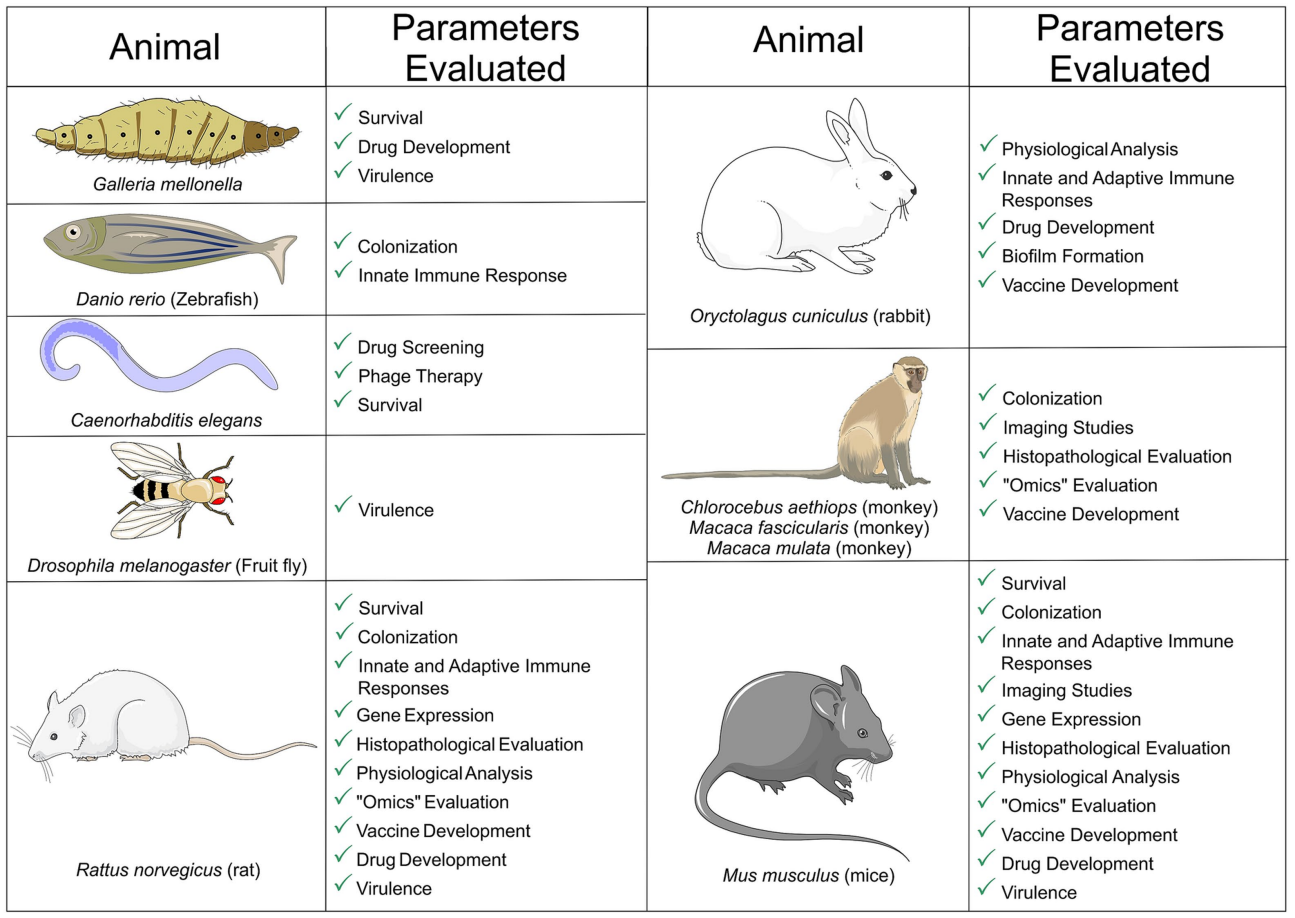
Animal models of *Klebsiella pneumoniae* mucosal infections. The main infection outcomes are shown for each model. The figure was partly generated using Servier Medical Art, provided by Servier, licensed under a Creative Commons Attribution 3.0 unported license. This figure was partly generated using templates from Servier Medical Art (Servier), licensed under a Creative Commons Attribution unported license.

Overall, when considering the diversity of host niches that *K. pneumoniae* can infect and the overall disease burden, animal models are an important tool to elucidate infection mechanisms and develop new, safer therapeutic and prophylactic strategies against this pathogen. The present review focused on the *in vivo* platforms deployed to evaluate the pathogenicity of *K. pneumoniae* during infection of the main mucosal sites (gastrointestinal, respiratory and genitourinary). We explored the methodologies used to establish infection and the results achieved in each animal model. Complex vertebrates with anatomical features and immune response similar to ours, like rodents and primates, were compared with simpler models, such as invertebrates and zebrafish, considering specific disease outcomes, comparative virulence, and host defense mechanisms (Figure 2). The different techniques were compared regarding complexity, requirement for specific equipment, relevance of the results and applicability to humans, and the more robust techniques were highlighted in each case.

Finally, we reviewed the current models used to evaluate the host-pathogen interactions during disease, and the efficacy of therapeutic agents and/or potential vaccine candidates against infections by *K. pneumoniae*.

This review includes papers written in English, chosen using the keywords correlated to the topics “animal models,” “*Klebsiella pneumoniae*” and “mucosal infection,” with an emphasis in articles published within the last 10 years in journals indexed in the PubMed database. Approval by animal research ethics committee was also used as a selection criterium.

## Animal models of infection by *Klebsiella pneumoniae*

### Animal models of *Klebsiella pneumoniae* respiratory infections

Hospital or community-acquired *K. pneumoniae* respiratory infections represent a serious public health threat, causing high mortality due to the common development into bacteremia (Antimicrobial Resistance Collaborators, 2022; Chen et al., 2022). *K. pneumoniae* is one of the main causes of pneumonia worldwide as a result from widespread colonization (Martin and Bachman, 2018; Chen et al., 2022). In some countries, the reports of *K. pneumoniae* community-acquired pneumonia cases which progressed to bacteremia, has surpassed those caused by *Streptococcus pneumoniae*—the leading cause of bacterial pneumonia (Lin et al., 2010).

The main models used to investigate the hallmarks of respiratory infections by *K pneumoniae* are mouse and rat, with a few studies being conducted in other mammals such as rabbits and monkeys. The different models of respiratory infections (summarized in Table 1) will be discussed individually in the next sections.

**Table 1:** *In vivo* models of respiratory infection by *Klebsiella pneumoniae.*

Inoculation method	Endpoint	Other analysis	*K. pneumoniae* strains	Type of animal model used	Animal strains	References
Intranasal inoculation	Survival; Bacterial burden in lungs and blood	Cytokine expression; Histological evaluation	Clinical isolates (common and hypervirulent type), ATCC 43816, ATCC 10031	Mice	BALB/C, C57BL/6J, CD-1, Swiss, ICR, C3H/HeJ, MF1, Kunming	Geller et al. (2018), Wieland et al. (2011), Kumar et al. (2020), Wolff et al. (2021), Russo et al. (2015), Jain et al. (2015), Yoshida et al. (2001), Branger et al. (2004), Mabrook et al. (2022), Li-Juan et al. (2022), Lavender et al. (2005), Meijer et al. (2021), Dong et al. (2021), Otto et al. (2021), Zhang et al. (2021)
Intratracheal inoculation (surgical incision)	Survival; Bacterial burden in the lungs and blood	Cytokine and chemokine expression; NK, T and neutrophil cells migration; Changes in the respiratory microbiota	Clinical isolate (common type), ATCC 43816, ATCC 27736	Mice	C57BL/6, NIH/Swiss, BALB/C	Zeng et al. (2003), Xu et al. (2014), Zeng et al. (2005), Vieira et al. (2016), Barletta et al. (2012), Morinaga et al. (2019), Fagundes et al. (2012), Deng et al. (2004), Liu J. et al. (2022)
Intratracheal inoculation (without surgery)	Survival Bacterial load in the lungs, blood, liver, spleen	Cytokine expression; Inflammatory response levels; Cell recruitment; Histological evaluation; Bioluminescent imaging; Cytological evaluation	Clinical isolates (common and hypervirulent), ATCC 43816, Xen 39	Mice	C57BL6/J (WT and mutants with C57/BL6J background), ICR	Zhao et al. (2015), Griepentrog et al. (2020), Olonisakin et al. (2016), Zhang J. et al. (2019) Nikouee et al. (2021), Hu et al. (2020)
Intratracheal spraying	Survival; Bacterial burden in the lungs and liver	Inflammatory response-related gene expression; Cytokine levels	700,603, NTUH-K2044	Mice	C57BL/6Cnc, BALB/C	Gou et al. (2021), Zheng et al. (2022)
Retropharyngeal inoculation	Bacterial burden in the lungs and spleen	Cytokine and chemokine expression; Fitness gene expression during infection	KPPR1 (Rifampin-Resistant Mutant of ATCC 43816)	Mice	C57BL/6, Mutants generated from a C57BL/6 background	Bachman et al. (2015), Holden et al. (2016)
Laryngoscopy	Survival	Cross-protection with an inactivated whole-cell *A. baumanii* vaccine against *K. pneumoniae* challenge	Clinical isolate	Mice	C57BL/6 (WT, Rag1^−/−^ and Tlr4^−/−^)	Gu et al. (2021)
Gavaging	Number of animals infected; Bacterial burden in lungs		Clinical isolates	Mice	ICR:CD-1, Swiss	Cortés et al. (2002), Rouse et al. (2006)
Direct inoculation with a cannula following intubation	Survival; Bacterial burden in lungs and blood	Body temperature and weight	EMC2003 (ESBL variant of ATCC 43816), EMC2014 (KPC variant of ATCC 43816)	Rat	Sprague–Dawley, RP–AEur–RijHsd	van der Weide et al. (2020a,b)
Intubation	Survival	Histological evaluation; Cytokine and chemokine expression	46,114	Rat	Sprague–Dawley	Mei et al. (2017)
Inoculation with a catheter	Bacterial burden in the lungs	Histological evaluation; Cytokine and chemokine expression		Rat	Sprague–Dawley	Gu et al. (2022)
Intranasal inoculation	Bacterial burden in the lungs	Cytokine expression; Histological evaluation; Physiological signs; Transmission electron microscopy of the lung tissues	ATCC 1705	Rat	Wistar, Sprague–Dawley	Wang et al. (2022), Aljohani et al. (2022)
Left side-only pneumoniae by intubation	Survival; Bacterial burden in the lung and blood	Blood extravasation; Histological evaluation; Tissue weight	ATCC 43816	Rat	RP–AEur–RijHsd	Schiffelers et al. (2000, 2001b), Bakker-Woudenberg et al. (2001)
Inoculation with a catheter	Bacterial burden in the lungs and blood	Histological evaluation; Leukocyte recruitment; Inflammatory response-related gene expression; Inflammatory response markers	NCTC 5055, Clinical isolate	Rat	Wistar, Sprague–Dawley	Chhibber et al. (2004), Chhibber et al. (2003), Sun et al. (2006)
Intrabronchial instillation with a bronchoscope	Lung bacterial burden	Radiographical score; Histopathological score; Host transcriptome	Clinical Isolate (ST258)	Non-rodent animal model	Cynomolgus macaques (*Macaca fascicularis*)	Malachowa et al. (2019)
Direct inoculation in the pleural space	Empyema positive cultures	Gas, pH, glucose and lactic acid levels; Leukocyte count	Clinical isolate	Non-rodent animal model	White New Zealand rabbits	Shohet et al. (1987)
Pre-colonized endotracheal tubes	Hyperthermia in biofilm formation		Clinical isolates	Non-rodent animal model	White New Zealand rabbits	Palau et al. (2023)

#### Mouse infection models

In mice, the main method to induce pneumonia is the direct instillation of bacteria in the mice nasopharynx through the nasal cavity (Wieland et al., 2011; Geller et al., 2018). This model has the advantages of the absence of any surgical procedure, in addition to the easy and quick manipulation of the animal. An important factor to consider in this type of infection is the volume of bacteria applied to the mouse. Lower volumes such as 5–10 μL can carry little to none of the inoculum into the lungs and usually promote only local nasopharyngeal colonization. To cause pneumonia, volumes between 25 and 50 μL are necessary, coupled with anesthesia to allow aspiration into the lungs. Interestingly, higher volumes do not appear to enhance lung delivery (Southam et al., 2002) and increase the risk of death by suffocation. Several different mouse strains have been tested using this inoculation model, including BALB/c (Kumar et al., 2020), C57BL/6 J (Wolff et al., 2021), CD-1 (Russo et al., 2015), Swiss (Jain et al., 2015), ICR (Yoshida et al., 2001), C3H/HeN and C3H/HeJ (parental toll-like receptor 4 deficient strain) (Branger et al., 2004), MF1 (Mabrook et al., 2022) and Kunming mice (Li-Juan et al., 2022). Similarly, there was also a great variety in the methods of anesthesia, which included, halothane (Lavender et al., 2005), isoflurane (Meijer et al., 2021), ketamine (Dong et al., 2021), ketamine and medetomidine (Otto et al., 2021) or xylazine mix (Zhang et al., 2021). A pneumonia model using direct bacterial instillation was established to evaluate the yersiniabactin receptor FyuA as a recombinant vaccine candidate against respiratory *K pneumoniae* infections in BALB/c mice. In the study, the authors used 10^3^ colony forming units (CFU) of a K2 isolate, American Type Culture Collection (ATCC) 43816 diluted in 50 μL. The control group showed an increased bacterial burden in the lungs, 48 h following infection, as well as higher levels of the inflammatory cytokines interleukin 17 (IL-17), tumor necrosis factor α (TNF-α) and interleukin 1β (IL-1β), oxidative stress markers myeloperoxidase (MPO) and nitrous oxide (NO), and histological damage, while recombinant FyuA immunized mice exhibited increased levels of interleukin 6 (IL-6) and interleukin 12 (IL-12), with a reduced bacterial burden in the lungs, 48 h following infection (Kumar et al., 2020). A similar methodology was applied to C57BL6/J mice, using the same bacterial strain (ATCC 43816) to analyze changes in the lung and gut microbiome following respiratory infection by *K. pneumoniae* and *Streptococci*. An early production of TNF-α was detected in the mouse lungs, followed by lung colonization at 12 h and dissemination to the bloodstream at 18 h post infection. These changes were accompanied by a shift in bacterial distribution in the lungs, with an increase in *Klebsiella* and decrease in *Streptococci*.

Another method of inoculation of bacteria is directly in the trachea, via a small surgery, whereas an incision exposing the trachea allows the inoculum, with volume ranging from 25 to 30 μL, to be delivered with aid of a 26- or 30-gage needle (Zeng et al., 2003; Xu et al., 2014). The mouse lineage most frequently used is C57BL/6 background (Zeng et al., 2003, 2005; Barletta et al., 2012; Xu et al., 2014; Vieira et al., 2016; Morinaga et al., 2019), although NIH/Swiss (Fagundes et al., 2012) and BALB/c mice have also been tested (Deng et al., 2004; Xu et al., 2014; Liu J. et al., 2022). Anesthesia was performed either with a xylazine-ketamine mix (Zeng et al., 2003, 2005; Barletta et al., 2012; Fagundes et al., 2012; Xu et al., 2014; Vieira et al., 2016) or isoflurane (Morinaga et al., 2019). Following the procedure, the incision is closed with surgical staples or Vetbond (Rosen et al., 2015). One example of pneumonia induced by intratracheal bacteria inoculation is the work conducted by Zeng and colleagues (Zeng et al., 2003), in which infection was caused by intratracheal delivery of 10^3^ CFU of the ATCC 43816 strain in 30 μL of final volume, through a 26-gage needle. The authors compared the immune response in control mice versus those previously injected with a recombinant, Ad5 human adenovirus-based platform expressing the macrophage inflammatory protein-1 alpha complementary DNA. The latter exhibited recruitment of neutrophils and activated NK cells to the lungs, as well as increased expression of gamma interferon (IFN-γ), which correlated with reduced bacterial counts in the lungs and bloodstream (Zeng et al., 2003). However, tracheal inoculation is possible without the need of surgical procedures. The suspension can be given straight to the lungs of the anesthetized animals by placing a metallic cannula with an inner tube, at the opening of the left bronchus, with 20 μL of volume injected with a micro-injection syringe. The bacteria are suspended in melt agar medium, which serves as an infection enhancer, providing a protective milieu for the inoculum. This method allowed the stable colonization of mice and rat lungs by multiple pathogens, including *K. pneumoniae* (Hoover et al., 2017). However, the authors conclude that this model is not suitable for evaluating survival as an endpoint, but rather should be used to determine CFU counts in the lungs. Intratracheal spraying was also performed to induce pneumonia in BALB/c mice anesthetized with pentobarbital. The mice were given 25 μL of *K. pneumoniae* 700603, at a concentration of 3 × 10^9^ (Gou et al., 2021). Similarly, *K. pneumoniae* NTUH-K2044 (hypervirulent K1 strain, 2 × 10^4^ CFU) was aerosolized and given in the tracheal bifurcation of female C57BL/6Cnc mice anesthetized with pentobarbital (Zheng et al., 2022). This infection model using the same hypervirulent strain was lethal for the mice within 48 h of challenge. The transcriptome of the infected animals showed an increased expression of genes related to acute inflammatory responses after 12 h of infection, with migration of granulocytes, monocytes and macrophages (Zheng et al., 2022).

*K. pneumoniae*-induced pneumonia can also be achieved with retropharyngeal inoculation. In this model, the inoculum was administered in C57BL/6 J mice, after isoflurane anesthesia (Bachman et al., 2015; Holden et al., 2016). This pneumonia model was used to evaluate the contribution of siderophore secretion for lung inflammation and bacteria dissemination during infection. A mutant Δ*tonB* strain that secretes, but does not import the siderophores, induced the release of IL-6 and the chemokines CXCL1 and CXCL2 in lung homogenates, colonized the lungs and spread to the spleen. Comparatively, mutant strains lacking different combinations of siderophores (enterobactin, salmochelin, and yersiniabactin), showed mixed results. Overall, the authors propose that siderophores play an essential role in the pathogenicity of *K. pneumoniae* respiratory infections, via interference with the immune system (Holden et al., 2016).

Another delivery mechanism is via laryngoscopy. Following pentobarbital anesthesia, the animal is positioned in a 45-degree angle and with aid of a laryngoscope blade, the trachea is exposed, and the bacteria are injected with a soft-end needle (Gu et al., 2021). Using the method developed by Spoelstra et al. (2007), a vaccine based on inactivated *Acinetobacter baumanii* was able to cross-protect against *K. pneumoniae* pneumonia, following intranasal challenge. Using the same pneumonia model, an inactivated, whole cell vaccine based on a *K. pneumoniae* isolate also reduced the death toll in the immunized mice (Gu et al., 2021).

A gavage or feeding needle can also be used to inoculate and establish the infection. This model was carried out in ICR:CD-1 (Swiss background) and Swiss mice, where 10^6^ to 10^7^ CFU of *K. pneumoniae* was given in 30–50 μL of volume directly in the trachea, through the blunt-end needle, in anesthetized animals (Cortés et al., 2002; Rouse et al., 2006). Following the inoculation of 10^7^ CFU of an extended-spectrum beta lactamase (ESBL) and non-ESBL producing *K. pneumoniae* strains, the animals were treated with different cephalosporins and the ability of the drugs to prevent colonization was evaluated. In the group challenged with the ESBL-producing strain, neither of the antimicrobials were capable to prevent colonization, while in the non-ESBL group, antibiotic treatment blocked colonization in some animals (Rouse et al., 2006).

It is also possible to deliver bacteria directly in the trachea by positioning the pipette tip above the mouse vocal cords (Zhao et al., 2015; Olonisakin et al., 2016; Griepentrog et al., 2020). The authors used a 200 μL tip to deliver an inoculum of 10^3^ CFU of ATCC 43816 in the colonization experiments (10^4^ in the lethal model) in wild-type C57BL6/J and the isogenic, thrombospondin-1 negative mutants. The work demonstrated that in the mutant group, the animals survived longer, with a reduced bacterial burden in the lungs and spleen and had a lower pulmonary histopathology score. Cytokine and MPO expression levels were also reduced (Zhao et al., 2015).

Similarly, the tongue pull technique can be used to reach the trachea. With aid of a forceps, the tongue is pulled out and the bacteria are inoculated in the trachea of the anesthetized animal (Zhang G. et al., 2019). This technique was used to deliver 50 μL of the ATCC 43816 strain to C57BL6/J mice. The animals treated with imipenem in combination with andrographolide sulfonate (an anti-inflammatory agent), showed 100% survival rate after challenge, while also reducing the CFU count in the lungs, with controlled inflammation and reduced lung tissue damage parameters (Zhang G. et al., 2019).

An otoscope can also be used to deliver the bacteria into the lungs (Nikouee et al., 2021). This is an interesting technique, since other methods such as tracheostomy can be stressful and induce bleeding and inflammation in the animal (Thomas et al., 2014). With the aid of the otoscope, a catheter was positioned in the trachea to deliver the inoculum to anesthetized ICR mice under immunosuppression with cyclophosphamide. The study intended to conduct a real-time monitoring of infection using a bioluminescent *K. pneumoniae* strain and found that the group treated with the highest immunosuppressor dosage presented increased bioluminescence in the lungs and tissue damage (Hu et al., 2020).

This method was also applied to male C57BL6/J mice, using 3 × 10^7^ CFU of the ATCC 43816 strain to evaluate the role of Beclin-1, an autophagy initiation factor, in pneumosepsis. Overexpression of Beclin-1 resulted in increased autophagy activation and reduced the burden, inflammation and tissue damage during infection, in comparison with the wild-type animals (Nikouee et al., 2021).

Although some of the endpoints varied among the studies, the results suggest that different methods of inoculation promote lung disease with inflammatory infiltrates and increased bacterial loads, and therefore may be used to investigate *K. pneumoniae* infection. An important aspect to be considered is bacterial dissemination from the lungs to other sites—a situation that is frequently observed in humans and is associated with poor prognosis. When considering the reviewed studies, invasiveness is majorly associated with the virulence and dose of the strain used to cause infection, and less a result of the inoculation method. Hypervirulent strains possessing multiple virulence factors, such as KPPR1-derived strains (including ATCC 43816) are highly lethal to mice and provide an interesting platform for investigating disseminated disease. However, since they kill the mice fast, these strains are usually not ideal for evaluating adaptive immune responses. Classical strains, on the other hand, are rapidly cleared from mice and are not suitable for survival studies (Russo et al., 2018, 2021).

A limitation common to all mouse studies is that mice do not die of bacterial pneumonia, but from disseminated infection with extremely high bacterial loads in the blood and major organs. Human patients, on the other hand, may present much lower bacterial counts and perish from the intense inflammatory responses associated with infection.

#### Rat infection models

In rats, pneumonia models were established either bilaterally or on the left side-only. For the induction of pneumonia in both lungs, Sprague–Dawley or RP–AEur–RijHsd rats were used. The animals were anesthetized with either isoflurane or medetomidine, intubated, immobilized vertically, and inoculated with 60 μL of ESBL and carbapenemase-producing *K. pneumoniae* isolates (KPC) (van der Weide et al., 2020a,b) to test the efficacy of antibiotic treatment. The use of tigecycline prevented the death of all animals against KPC-induced pneumonia/septicemia, while the group treated with meropenem was not protected. Meanwhile, in the ESBL group, all animals survived when treated with meropenem (Van der Weide et al., 2020b). Another study used tracheal instillation with 2.4 × 10^8^ CFU of strain 46,114 of *K. pneumoniae* to induce pneumonia in Sprague–Dawley rats anesthetized with chloral hydrate. By employing this inoculation method, the effects of the Dusuqing granules, a compound based on an herb used in Chinese traditional medicine, was evaluated in the lung inflammatory process caused by *K. pneumoniae* infection. The treatment was able to reduce the tissue inflammation and damage. Cytokine and chemokine levels were reduced in the lungs of the treated animals, as were the leukocytes in the blood and the bronchoalveolar lavage fluid The authors hypothesize that the anti-inflammatory modulation is related to the downregulation of the NF-κB/MAPK signaling pathway (Mei et al., 2017).

An alternative delivery method in rats is the use of a 22-gage catheter intranasally, through the trachea, in which 50 μL of the *K. pneumoniae* CMCC (B) 46,117 suspension was given to Sprague–Dawley rats (Gu et al., 2022). Using the catheter to deliver the bacterial load, the pneumonia model was employed to investigate the effects of the coadministration of azithromycin with a traditional Chinese medicine formulation. The antibiotic mix improved the bacterial clearance and reduced inflammation parameters (Gu et al., 2022) Direct instillation of the bacterial suspension is also possible in rats. To establish an exudative pneumonia model of *K. pneumoniae*, Wistar male rats were given 50 μL of ATCC 1705 intranasally, in combination with lipopolysaccharide (LPS) administered intraperitoneally daily, for 5 days. This model was used to evaluate the effects of a traditional Chinese medicine formulation in the prophylaxis or treatment of lung infection. While the compound did not show direct antibacterial activity, it reduced the CFU counts in the bronchoalveolar lavage fluid (BALF) and histological damage in the lungs, while improving physiological parameters. Seric IL-6 and alveolar lavage IL-1β cytokine expression was also reduced in the treated group. While the treatment did induce some enhancement, the pre-treatment showed no effect in the pneumonia establishment (Wang et al., 2022). Similarly, female Sprague–Dawley rats anesthetized with isoflurane received 50 μL of an inoculum containing 10^7^ CFU of a multi-drug resistant clinical strain with similar volumes in both nares. The authors used a zinc oxide nanoparticle in combination with sulphadiazine, delivered through aerosolization, to evaluate protection against pneumonia. After 4 days, the bacterial counts in the lungs were reduced and the histopathological signs of inflammation tissue damage were diminished (Aljohani et al., 2022).

Left side pneumonia models were established in female RP–AEur–RijHsd rats, where the animals were anesthetized with fluanisone and fentanyl, then pentobarbital. After the procedure, the left bronchus was intubated, and a bacterial load of 10^6^ CFU of *K. pneumoniae* strain 43,816 were administered to the left lung, diluted in 200 μL of saline (Schiffelers et al., 2000, 2001a,b; Bakker-Woudenberg et al., 2001). The model was used to demonstrate the kinetics of the deposition and the effect of polyethylene glycol coating of liposomes in the target sites. Lung inflammation induced by *K. pneumoniae* infection resulted in enhanced liposomal deposition, possibly through increased capillary permeability in the inflamed tissue (Schiffelers et al., 2000).

Lobar pneumonia has also been successfully induced in rats using a catheter surgically inserted through the trachea. In female Wistar rats, 10^7^ CFU of *K. pneumoniae* National Collection of Type Cultures (NCTC) 5055 (K2/O1 strain) in 1 mL of final volume was given straight to the lungs, following an incision in the rat’s trachea, while the animal was anesthetized with pentobarbital, and immobilized in a supinated position (Chhibber et al., 2003, 2004). One of the applications of this model was the use of LPS, or liposomes with a LPS coating, as a vaccine against *K. pneumoniae* pneumonia. Both the pure LPS and the LPS-coated liposomes were able to reduce the bacterial burden in the lungs (Chhibber et al., 2004). A similar model was applied in Sprague–Dawley rats, whereas 200 μL of the inoculum (1.3 × 10^8^ CFU of an ESBL-producing, clinical isolate) was administered with a 26-gage needle, following the exposal of the trachea. The anesthesia was induced with ketamine. This model was used to demonstrate the effects of the exposure of the animals to different concentrations of nitric oxide and oxygen in the treatment of *K. pneumonia*-induced pneumonia. Varied combinations of nitric oxide and lowered oxygen concentration were shown to reduce the total bacterial count in the lungs and blood, while diminishing the expression of the proinflammatory cytokines TNF-α and intercellular adhesion molecule 1 (ICAM-1) expression levels (Sun et al., 2006). In summary, rats have been used as a model for *K. pneumoniae* pneumonia mainly to evaluate the efficacy of antibiotic treatment and experimental vaccines. One advantage of rats is the possibility of inducing lobar pneumonia, as observed in many human patients, especially in community-acquired respiratory infections. Also, the larger size compared to mice facilitates direct access to the lower respiratory tract through surgery or cannulation, allowing the direct delivery of the inoculum. Larger size and increased weight may also be more suitable for evaluating adaptive immune responses in studies using survival as an endpoint, since rats tend to survive longer periods than mice after challenge. Nevertheless, rats require more space and increased maintenance costs when compared to mice and have fewer advocated methodologies for their study in *K. pneumoniae* infections.

#### Other animal models

Besides mice and rat models, pulmonary *K. pneumoniae* infection was also induced in cynomolgus macaques. In that study, 10^8^ and 10^10^ CFU of a carbapenem-resistant ST258 strain, were inoculated in 8–10 years old female primates with aid of a bronchoscope, delivering the bacterial load directly into the lungs. In both infection groups, the *K. pneumoniae* load was able to induce pneumonia, which was then used to test the potential protective effect of *K. pneumoniae* capsular polysaccharide (CPS) against respiratory infection. The CPS formulation reduced the bacterial burden in the lungs, inducing antibodies that promoted opsonophagocytic killing by polymorphonuclear leukocytes (PMN) *in vitro*. The advantage in the use of primates as a study subject is the closer similarities to humans when compared to rodents, sharing anatomical and immune features, while also maintaining the susceptibility to infection, which is considerably higher in mice (Malachowa et al., 2019). However, the manipulation and maintenance of the animals are more laborious and demanding when compared to small rodents. Only a few specialized animal facilities are equipped to house this type of animal.

An empyema model was established using male and female white New Zealand rabbits. Following the artificial creation of pneumothorax and pleural effusion, 10^9^ CFU of a *K. pneumoniae* clinical isolate in 1 mL, was given through a 16-gage cannula. This model was used to analyze the effects of gentamicin and oxygen administration in empyema, which showed that higher O_2_ levels can improve the infection prognosis (Shohet et al., 1987).

Another study used male, white New Zealand rabbits to investigate biofilm formation *in vivo*. The model consisted in the introduction of an endotracheal tube previously colonized with different biofilm-producing strains of *K. pneumoniae* or *P. aeruginosa* in the trachea of the rabbits, with a hyperthermia device. The goal was to evaluate the biofilm formation in a fever state, and with temporary 42°C pulses, biofilm formation was greatly, though not completely inhibited (Palau et al., 2023).

Rabbit models of respiratory infections can be an interesting approach, depending on the desired outcomes. In an FDA-backed study to validate new animal models, *Pseudomonas aeruginosa* was used to induce pneumonia and it was found to reproduce many of the typical hallmarks found in humans, such as tissue damage and inflammation, changes in gasometry values, blood pressure, and white-blood cell counts. Eventual infection metastasis was also observed, in some cases leading to death (Nguyen et al., 2021; Gras et al., 2023). However, there is still very limited research using rabbits as a model for *K. pneumoniae* infection, and further studies are needed to accurately determine how robust the model is in terms of reproducibility, evaluation of adaptive immune responses during infection, duration of colonization and survival.

In summary, different animal models have been used to successfully reproduce the hallmarks of *Klebsiella pneumoniae* respiratory infections, with mice being the most used species. However, most of the infection protocols still rely on artificial inoculation routes, involving tracheal incisions or catheters to deliver the bacteria. While these may replicate some of the infections occurring in hospital settings, they do not mimic the natural infection routes in the community.

When considering mouse models in respiratory infections, the direct intranasal inoculation of bacteria appears to be the most advantageous technique to study community acquired infections, since it can: (i) provide data on the different stages of disease pathogenesis as well as the contribution of specific virulence factors; including acquisition, transition to the lungs, establishment and dissemination; (ii) evaluate specific components of the adaptive immune response; (iii) provide reproducible results in survival/colonization without the need for highly specialized training and equipment; (iv) adequately assess the protective efficacy of therapeutic agents or vaccine candidates.

### Animal models of *Klebsiella pneumoniae* oral/gastrointestinal infections

Gastrointestinal infections are highly prevalent diseases worldwide, where the most frequent causative agents in these infections are Gram-negative bacteria that reside in the human intestine (Joseph et al., 2021), especially Enterobacteriaceae, including *K. pneumoniae* (Osbelt et al., 2021). Its prevalence in hospitalized patients ranges from 3 to 18% (Gorrie et al., 2017; Joseph et al., 2021), while colonization in healthy individuals varies from approximately 6% in Europe to 20% in Africa (WHO, 2017).

Analysis of rectal and throat swabs from patients admitted in intensive care units found that 6% of these patients were colonized with *K. pneumoniae*. Gut colonization on admission was significantly associated with subsequent infections, with 49% of *K. pneumoniae* infections being caused by a strain found in the patients’ microbiota (Gorrie et al., 2017), and possibly transmitted through the fecal-oral route (Young et al., 2020). Furthermore, antibiotic treatment reduces microbial diversity in the GI tract, causing dysbiosis which favors subsequent colonization with *K. pneumoniae* (Martin and Bachman, 2018; Chen et al., 2022). *K. pneumoniae* dysbiosis in the GI correlated with the establishment of systemic infections, such as pneumonia (Wu et al., 2020; Jiang et al., 2022) and liver abscess (Zheng et al., 2021), and it also contributes to inflammatory bowel diseases (Khorsand et al., 2022).

Colonization of GI tract by *K. pneumoniae* is a necessary step for infection (Buffie and Pamer, 2013; Gorrie et al., 2017). Therefore, developing models that mimic this route of colonization and infection is of great importance for understanding disease pathogenesis and for the development of effective therapeutic/preventive strategies. Most animal studies investigating *K. pneumoniae* GI colonization used mice, since they are small, easy to breed and maintain, and reproduce infection hallmarks observed in humans (Ferreira et al., 2018; Muggeo et al., 2018; Cassini et al., 2019; Young et al., 2020; Joseph et al., 2021), as discussed in the next section and compiled in Table 2.

**Table 2:** *In vivo* models or oral and gastrointestinal infections by *Klebsiella pneumoniae.*

Inoculation method	Endpoint	Other analysis	*K. pneumoniae* Strains	Type of animal model used	Animal strains	References
Water inoculation	GI tract colonization	Histological evaluation	Clinical isolates	Mice	C57BL/6J	Lau et al. (2008)
Intranasal inoculation	GI tract colonization		Clinical isolate	Mice	Swiss-Webster	Lau et al. (2008)
Oral administration	GI tract colonization; Oropharyngeal colonization; Fecal CFU count; Survival; Burden in the kidneys, liver and spleen	Histopathological evaluation; Cell proliferation and differentiation, Cytokine expression; Microbiome diversity; Serum LPS, bilirubin and alkaline phosphatase levels; Genotyping and expression levels; Body weight, Metabolomic analysis	Clinical isolates, B5055	Mice	BALB/c, C57BL/6, C57BL6/J, C57BL/6N, C57BL/6NTac, MRL/MpJ, CFW1	Osbelt et al. (2021), Young et al. (2020), Chiang et al. (2021), Liu J. Y. et al. (2022), Nakamoto et al. (2019), Kamata et al. (2020), Boll et al. (2012)
Gavaging	Survival; GI tract colonization; Burden in the spleen, liver and lungs; Fecal CFU count	Body weight; Histopathological evaluation; Gene expression; Microbiome diversity; Serum level of aspartate transaminase, alanine transaminase, triglycerides, thiobarbituric acid-reactive substances; Cell proliferation and differentiation; Echocardiographic parameters; Competition during colonization	Clinical isolates, ATCC BAA-2146, ATCC 10031, NTUH-K2044	Mice	C57BL/6, C57BL/6J, OF1, BALB/c, BALB/cByL, CF1, 129×1/SvJ	Young et al. (2020), Lau et al. (2008), Hennequin and Forestier (2009), Maroncle et al. (2006), Calderon-Gonzalez et al. (2023), Yuan et al. (2019), Kienesberger et al. (2022), Hsieh et al. (2010), Hsu et al. (2019), Perez et al. (2011), Han et al. (2023), Lagrafeuille et al. (2018)
Contamination of the water	GI tract colonization; Fecal CFU count	Microbiome diversity; *In situ* hybridization	Clinical isolates	Mice	C57BL/6	Favre-Bonté et al. (1999), Le Guern et al. (2019)
Nasogastric tube insertion	Fecal CFU count		Clinical isolate	Rat	Wistar	Ruijs and van der Waaij (1986)
Gavaging	Fecal CFU count	Microbiome diversity; Genotyping	Clinical isolate	Rat	Sprague–Dawley	Ye et al. (2019)
Contamination of the water	Burden in the heart, intestine, spleen and liver	Neutrophil recruitment; Macrophage phagocytic ability	Clinical isolates	Non-rodent animal model	Zebrafish	Zhang X. et al. (2019)
Injection in different sites of the mouth cavity	Abscess and inflammatory infiltration	Histopathological evaluation; S-IgA and IgG secretion in serum and feces	Clinical isolate	Non-rodent animal model	Chinchilla rabbits	Marinova et al. (2000)

#### Mouse infection models

The best described methods of inoculation are through gavaging (intragastric administration) or direct oral administration. When the bacterial suspension is given orally, pretreatment with a large-spectrum antibiotic (or a cocktail) is usually performed in the days or weeks preceding challenge to deplete the mouse gut microbiota (Maroncle et al., 2006; Lau et al., 2008; Hennequin and Forestier, 2009). The described volumes of the bacterial suspension vary between 100 and 200 μL. BALB/c (Chiang et al., 2021), C57BL/6 (Nakamoto et al., 2019; Sequeira et al., 2020; Liu J. Y. et al., 2022), C57BL6/J (Young et al., 2020), C57BL/6 N (WT and Rag2 ^−/−^) and C57BL/6NTac (Osbelt et al., 2021), MRL/MpJ (Kamata et al., 2020) and CFW1 (Boll et al., 2012) mouse strains have been used in colonization experiments.

Young and colleagues have described a mouse model of GI infection where the animals received the bacteria orally, diluted in a 2% sucrose solution, delivered through a pipette tip, without previous antibiotic treatment. The authors also induced the infection via gavage, with a 20-gage feeding needle (Young et al., 2020). Administration of at least 10^5^ CFU of *K. pneumoniae* was enough to colonize the GI tract and oropharynx, regardless of the infection route. This model can be applied as an alternative to the standard infection protocols where the administration of antibiotics prior the colonization leads to a disruption on the native microbiota allowing *K. pneumoniae* to stablish colonization (Young et al., 2020). It could also be used to study community acquired *K. pneumoniae* infections.

The use of a gavage needle allows direct instillation in the lower GI tract; however, to neutralize the stomachal acidity, a sodium bicarbonate treatment (0.2 M in 200 μL) is performed 5 min prior to the inoculation, resulting in a successful colonization with 100 μL of the bacterial suspension (Calderon-Gonzalez et al., 2023). This model was applied in different mice strains, including C57BL/6 (Calderon-Gonzalez et al., 2023), C57BL/6 J (Lau et al., 2008; Yuan et al., 2019; Young et al., 2020; Kienesberger et al., 2022), OF1 (Maroncle et al., 2006; Hennequin and Forestier, 2009), BALB/c (Hsieh et al., 2010), BALB/cByL (Hsu et al., 2019), CF1 (Perez et al., 2011) and 129 × 1/SvJ (Han et al., 2023).

A work published by Lagrafeuille and colleagues investigated the use of probiotic bacteria as treatment against *K. pneumoniae* infections. They found that the addition of the cell-free supernatant from *Lactobacillus plantarum* impaired biofilm formation by *K. pneumoniae in vitro*. However, when tested in mice, the group infected with both bacteria presented a longer persistence of *K. pneumoniae* when compared with the control group (infected with *K. pneumoniae* only) (Lagrafeuille et al., 2018). This result reinforces the importance of having *in vivo* models to confirm the *in vitro* findings.

Another technique employed to colonize the gut microbiota of mice is through “contamination” of the drinking water with the intended bacteria. In the works describing the method, the animals received preventive antibiotic treatment (also in the water) with either clindamycin or streptomycin, followed by administration of water containing the bacteria (Favre-Bonté et al., 1999; Le Guern et al., 2019). Aiming to demonstrate the dynamics between GI infection and antibiotic administration, male C57BL/6 mice received 10^7^/mL of a New Delhi metallo-beta-lactamase-1 (NDM-1) positive strain in the water, after antibiotic treatment. The authors propose a protocol for the use of the antimicrobial where, based on the timing of the colonization process, clindamycin treatment a week before the inoculation showed the best results in the colonization model. When clindamycin was given 2 or 3 weeks before or after the inoculation, gut colonization was not as successful (Le Guern et al., 2019).

Perez and colleagues used a similar model to determine the effect of the antibiotic treatment on GI colonization, establishment, and elimination by *K. pneumoniae*. The animals received antibiotic treatment by subcutaneous injection every day during 8 days; on day 3, they were infected with *K. pneumoniae* and the occurrence of colonization was monitored through fecal analysis (Perez et al., 2011). The levels of *K. pneumoniae* in the feces remained high during antibiotic treatment, but fell gradually afterwords until clearance, suggesting that the reduction of the commensal microbiota by antibiotic therapy favors GI colonization by *K. pneumoniae* (Perez et al., 2011). In humans, although many normal microbiota are not resistant to *K. pneumoniae* colonization, antibiotic treatment can further contribute to *Klebsiella pneumoniae* infection, which often occurs at the hospital environment. The inhibition of exogenous colonization can be attributed either to competition among the microbes or the interaction of the microbiota with mucosal immune defenses, intensifying its response against invaders (Buffie and Pamer, 2013).

A study by Osbelt et al. using germ-free mice transplanted with a human microbiota evaluated stool samples from healthy individuals (adults and kids) and applied an *in vitro* screening to identify the microbiota composition looking for microorganisms present in the healthy donors that could eliminate *K. pneumoniae* colonization. They identified the commensal bacterium *Klebsiella oxytoca* as able to strongly reduce colonization by *K. pneumoniae* (Osbelt et al., 2021). *K. oxytoca* was shown to cooperate with other commensal bacteria to displace *K. pneumoniae* from the GI tract, which could potentially be applied as a probiotic treatment to prevent *K. pneumoniae* infections during hospitalization.

#### Rat infection models

In female Wistar rats, the GI tract colonization was obtained with an intragastric tubing, inserted surgically through the esophagus, following chloral hydrate anesthesia, with an inoculum of 1 mL containing a mixture of neomycin-resistant *Escherichia coli* and *K. pneumoniae*. In the co-colonization model, the infection lasted longer in animals that received the neomycin treatment when compared to the group who received the bacteria and the irrigation fluid only, without the antimicrobial agent (Ruijs and van der Waaij, 1986). A different instillation approach of the bacteria is via gavage, where 2 × 10^9^ CFU of bacteria diluted in 1 mL was administered to male Sprague–Dawley rats, following treatment with an antibiotic cocktail. Both the bacterial load and the antimicrobials were administered through gavage. *K. pneumoniae* was recovered from stool samples up to 16 days after challenge. The study described how the use of different types of antimicrobials can influence in the transmission of resistance genes via plasmids in the rat GI tract (Ye et al., 2019).

Very few studies have focused on animal models to investigate oral mucosal infections by *K. pneumoniae.* A screening of *K. pneumoniae* strains displaying the HMV phenotype was conducted in African green monkeys, rhesus and cynomolgus macaques, tested via polymerase chain reaction (PCR) of oropharyngeal and rectal swabs. Most of the *K. pneumoniae-*positive cultures were negative for the HMV phenotype, while 19 of the 307 animals tested positive for HMV-positive strains. The work demonstrated that *K. pneumoniae* is able to colonize the oral microbiota in non-human primates, most likely through the fecal-oral route (Burke et al., 2009).

Another study investigated the protective potential of Dentavax, a formulation based on inactivated *K. pneumoniae*, *Streptococcus pyogenes*, *Staphylococcus aureus*, *Candida albicans*, and *Lactobacillus acidophilus* strains. Chinchillas were immunized and challenged with 200 μL (5 × 10^9^ CFU/mL) of bacterial suspensions containing all the bacterial species in the formulation, delivered with injections made with a tuberculin syringe, in six different points of the oral cavity. The immunized animals showed a faster recovery, with fewer inflammatory histological signs. In the peripheral blood of the immunized animals, increased phagocytic activity of polymorphonuclear leukocytes were observed, while the sera showed immunoglobulin G (IgG) production against the bacteria included in the vaccine. Also, specific secretory IgA (S-IgA) production was detected in the feces (Marinova et al., 2000).

As mentioned for respiratory infection models, rodents are the preferred animals to evaluate *K. pneumoniae* GI infections. Several inoculation techniques have been employed to successfully establish colonization. However, an important issue regarding GI models is the presence of a natural microbiota that greatly impacts the infection outcomes. An alternative to surpass this limitation is the use of animals with a humanized microbiota, that allows the evaluation of bacterial interactions during infection, mimicking the conditions found in the human host. Another important aspect of GI infections is the effect of previous antibiotic treatment on dysbiosis, that leads to *K. pneumoniae* infections in humans; this can also be evaluated using mice with a humanized microbiota, providing important insights for future antibiotic treatment.

In summary, considering the differences between the mouse GI microbiome—which is naturally resistant to *K. pneumoniae* colonization—and the human microbiome, a more permissive environment for *K. pneumoniae*, an ideal model would employ animals transplanted with human bacteria. The oral route of administration is preferable because it better mimics the natural acquisition of the pathogen and allows the investigation of host defense mechanisms activated during the bacterial transition from the oral cavity to the lower GI tract. However, when considering specific infections such as liver abscess, a more artificial route, such as intraperitoneal injection may be used (Wu et al., 2022). Though, depending on the invasiveness of the strain, oral inoculation can also lead to infection in other sites as liver and spleen (Hsieh et al., 2012).

### Animal models of *Klebsiella pneumoniae* urinary tract infections

Urinary tract infections (UTI) are among the most common infections in the community and in hospital settings. An UTI can progress to pyelonephritis, kidney damage and sepsis. Bacteria are the main causative agents of UTI, including Gram-positive and Gram-negative pathogens, with the most prevalent being *Escherichia coli* and in second position, *Klebsiella pneumoniae*. Both are commonly identified in infections of variable severity (Flores-Mireles et al., 2015). A prediction using statistical models showed that just in the year of 2019, around 65,000 deaths were credited to urinary infections caused by multi-drug resistant bacteria. With another 200,000 deaths indirectly related to such infections (Li et al., 2022). Another reason of concern is the increasing rates of antimicrobial resistance in UTIs, which limit the therapeutic choices and worsen the disease burden, reinforcing the urgent need for new therapies and prophylactic strategies (Flores-Mireles et al., 2015).

In that sense, animal models are important for studying the host-pathogen interactions during urinary tract infections, and for testing new antibiotics/vaccines. As described for other mucosal infection models, most animal studies evaluating UTI are performed in mice, which are a better option than rats, as they have a greater number of glycolipid receptors in the urethral tissue, thus promoting better adhesion. Furthermore, the bladders of mice and humans share conserved proteins named uroplakins, which play a role in fimbriae-mediated bacterial adhesion (Murray et al., 2021).

The next section includes the animal models used to study UTIs. These studies are summarized in Table 3.

**Table 3:** *In vivo* models of urinary tract infections by *Klebsiella pneumoniae.*

Inoculation method	Endpoint	Other analysis	*K. pneumoniae* strains	Type of animal model used	Animal strains	References
Transurethral catheterization	Burden in the bladder, kidneys, spleen and liver; Urine CFU count	Histopathological evaluation; IBC quantification; Colonization competition; Body weight	Clinical isolate, KPPR1, NTUH-K2044	Mice	C3H/HeN, C3H/HeJ, BALB/c, CBA/J	Rosen et al. (2008a,b,c), Gomes et al. (2020), Saenkham et al. (2020), Mason et al. (2023)
Transurethral inoculation with urethral tubing	Burden in the bladder	CFU count of the catheter	Clinical isolate	Mice	C57BL/6NCr	Murphy et al. (2013)
Transurethral catheterization	Burden in the bladder and kidneys		Clinical isolate	Rat	Sprague–Dawley	Reid et al. (1985)
Transurethral catheterization	Burden in the bladder, kidneys; Urine CFU count		Clinical isolates, ATCC 10031	Rat	Wistar (CFHB)	Camprubí et al. (1993), Regué et al. (2004)

#### Mouse infection models

The most described method of bladder inoculation is via a catheter inserted directly through the urethra. Anesthetized animals are placed horizontally and catheterized. After the insertion, with the aid of a syringe, the bacterial suspension or treatment is inoculated into the lower urinary tract (bladder) of female mice (Thai et al., 2010).

Many different mouse strains have been used to study *K. pneumoniae* urinary infections, including C3H/HeN (Rosen et al., 2008a,b), C3H/HeJ (Rosen et al., 2008c), BALB/c (Gomes et al., 2020) and CBA/J (Saenkham et al., 2020; Mason et al., 2023). Anesthesia is usually performed using methoxyflurane (Rosen et al., 2008a,b,c) or a ketamine and xylazine mixture (Gomes et al., 2020). A study evaluating the association between diabetes and urinary tract infections inoculated C3H/HeN, C3H/HeJ and C57BL/6 mice with different bacterial strains, including a cystitis isolate of *Klebsiella pneumoniae* TOP52 1721, using a transurethral catheter. After the infection period, the bladder and kidneys were aseptically removed for bacterial counts. The TOP52 1721 strain was able to cause infection in the bladder and kidneys of mice with diabetes at higher titers than in healthy mice, indicating an increased susceptibility to UTIs in that group (Rosen et al., 2008c).

A study characterized the molecular difference of FimH of a uropathogenic *E. coli* (UPEC) isolate UTI89 and *Klebsiella pneumoniae* cystitis isolate TOP52. Strains used for the urinary tract infection model were UTI89 cystitis isolate UPEC; UTI89 Δ*fimH* (mutant strain lacking the adhesin FimH); TOP52 1721, a *K. pneumoniae* cystitis isolate; and TOP52 Δ*fimK* (mutant strain lacking the fimbriae regulator FimK). The infection was induced by inoculating 50 μL of a 1 to 2×10^7^ CFU suspension the strains via the urethra, into 8-week-old female C3H/HeN mice, and to perform the quantification of bacteria present in the animals’ tissues, the bladder and kidneys were collected aseptically after 6 h, 1 and 14 days, and plated. The bacterial titers in the bladder were higher at all timepoints for *E. coli* UTI89 strain (which had fimbriae) when compared to UTI89 Δ*fimH* (FimH mutant), which was also seen between the wild-type *K. pneumoniae* TOP52 strain, in comparison to the FimH-negative mutant. The bladder CFU counts of the UPEC group was higher than the *K. pneumoniae* group. However, 14 days post-infection, the recovered CFU was similar. Colonization of the kidneys was initially higher in the UPEC group but became similar to *K. pneumoniae* in later data points. Wild type and mutant *K. pneumoniae* strains showed equal titers in all measured times. The study demonstrated that for *K. pneumoniae*, FimH did not have a critical role in the initial steps of bladder infection, however it became required in later stages. For *E. coli* UTI89, FimH appears to play a more prominent role. Overall, the study shows the role that FimH in the bladder colonization and invasion, infection persistence and development of intracellular bacterial communities (IBCs) in both strains (Rosen et al., 2008b).

Using the same infection model, Rosen et al. evaluated the role of the FimK regulator in the urinary tract infection process. *K. pneumoniae* lacking *fimK* showed an increased type I fimbriae expression, which reflected in the increased CFU count in the bladder and kidneys, and IBC formation in the animal group infected with the mutant strain, when compared to the wild type. The authors propose that the downregulated type I fimbriae expression could partially explain how UTIs caused by *E. coli* infections are more common when compared to *K. pneumoniae* (Rosen et al., 2008a).

A study by Gomes et al., describing the transcriptional regulator of the *kpfR* gene cluster demonstrated that the regulator plays an important role in *K. pneumoniae* pathogenicity in urinary tract of mice. In the study, female BALB/c mice were inoculated via the transurethral route with a K2 clinical strain isolated from a patient diagnosed with UTI or its isogenic KpfR-negative mutant. The mutant, which exhibited a hyper fimbriated phenotype, displayed reduced ability to colonize the mouse bladder and was cleared faster than the wild-type strain. The authors suggest that overexpression of fimbriae in the mutant promotes a more robust immune response that leads to quick bacterial elimination by the host (Gomes et al., 2020).

A similar infection model used a silicone tube attached to a needle, which was inserted in the bladder through the urethra opening of C57BL/6NCr mice under isoflurane anesthesia. In this study, 50 μL of the 10^7^ CFU bacterial suspension were inoculated. Using this model, the role of type I and III *K. pneumoniae* fimbriae in the colonization of the urinary tract was evaluated in the presence (or absence) of the silicone implant in the bladder, which was used to simulate a urinary catheter. In the presence of the implant, colonization of the bladder was augmented, especially when the insertion had been performed 24 h prior to the inoculation. *K. pneumoniae* strains lacking the type I and type III fimbriae demonstrated a reduced ability to colonize the bladder in both the catheterized and un-catheterized groups at the 6- and 48-h endpoints. Finally, the number of bacteria recovered from the implants was lower in the group inoculated with the mutant lacking type III fimbriae, suggesting a bigger impact of this fimbriae in the colonization of the abiotic surface (Murphy et al., 2013).

The murine model of urinary tract infection was also used for assessing the impact of hyperglycosuria on bacterial colonization by *Klebsiella pneumoniae.* The excess glucose excretion was induced using dapagliflozin, a drug for controlling diabetes. The *K. pneumoniae strain* used was KPPR1 and the experiments were conducted on adult (4–6 weeks) female CBA/J mice. The infection was induced by inoculation of 10^8^ CFU of the KPPR1 strain into the bladder via transurethral route. A syringe pump connected to a polyethylene tube, with a constant, low flow, was used to prevent leakage of the inoculum due to urinary reflux. Urine was collected at 6, 24, 48 h, or 7 days, and plated for bacterial count; the bladder, spleen and liver were removed aseptically and plated as well. A higher bacterial load was evident in both urine and bladder of the mice with hyperglycosuria. This group also exhibited a greater systemic spread as other organs, such as spleen and liver showed a higher bacterial load, indicating that high glucose levels promote UTI by *K. pneumoniae* (Saenkham et al., 2020).

In conclusion, mice have been extensively used as models of *K. pneumoniae* UTI, including local bladder infection and ascending pyelonephritis. In that regard, the increased vesical-ureteric reflux presented by the C3H/HeJ strain—a retrograde urine flow resulting from a congenital anomaly of the urinary tract (Murawski et al., 2010)—makes it an ideal model to investigate more complicated, ascending infections, like those observed in human patients with the same condition.

An important aspect that remains to be explored is the gender-related differences in UTI by *K. pneumoniae*. There are protocols available to induce infection in male mice using transurethral instillation (Zychlinsky Scharff et al., 2017) or surgically through an abdominal incision followed by direct bladder inoculation using a needle (Olson et al., 2016). Studies with *E. coli* have demonstrated that male mice develop more severe, chronic infections that are influenced by androgen exposure (Olson et al., 2016). Therefore, UTI studies using male mice are necessary to evaluate gender-related differences in *K. pneumoniae* infections.

#### Rat infection model

A few studies have used rats to investigate UTI by *Klebsiella pneumoniae*. In female Sprague–Dawley rats anesthetized with pentobarbital, bladder colonization was achieved with a catheter, in which, 50 μL of a 5 × 10^9^ bacterial suspension incorporated in agar beads were inoculated to the animals. Using this urinary infection model, the effectiveness of a treatment with *Lactobacillus casei* prior to the infection was evaluated. *L. casei* was also incorporated into beads and while it is not able to colonize the kidneys of the animals, the colonization of the urinary tract with *L. casei* prevented the installment of UTI and pyelonephritis in the animals who received the lactobacilli-coated beads (Reid et al., 1985). A similar method was used in Wistar (CFHB) rats, in which following anesthesia the abdominal area was massaged to expel the urine. Then, a urinary cannula was used to introduce the inoculum, in a final volume of 1 mL. When the inoculation step was finished, a clamp was used in the urethral meatus to prevent bacterial leakage and taken out after 10 min (Camprubí et al., 1993; Regué et al., 2004). This protocol was used to evaluate the role of the O-antigen and CPS in the UTI development. Strains lacking the O-antigen showed a substantial decrease in the ability to colonize the rat’s kidneys and bladder, whereas, in the infection model tested, the K-antigen did not appear to bear the same level of importance (Camprubí et al., 1993).

#### Other UTI animal models

An *ex vivo* porcine model was employed to induce a catheter-associated UTI. The model is based on a modified Foley catheter, which was introduced in the urethral tract of a euthanized female pig. With a silicone tube, the catheter was placed, then inflated to stay in place throughout the experiment. The catheter contained pre-formed 24 h bacterial biofilms, including a carbapenem-resistant *K. pneumoniae* strain. Following the introduction, the apparatus was irrigated with a combination of antibiotic solutions, then segmented for bacterial quantification. The animal’s urethra and bladder were also analyzed (Vargas-Cruz et al., 2019). The model reproduced the parameters of catheter associated infections in human patients, while showing that the antibiotic irrigation was able to reduce bacterial colonization of the urinary tract.

As discussed for respiratory and GI infections, mice have been used in most of the studies investigating UTI by *K. pneumoniae*, with transurethral injection using catheters being the main technique for bacterial inoculation. This inoculation route results in direct delivery of bacteria in the bladder, but it has a few limitations: the size and position of the mouse urethra make it hard to access; urine flow may contribute to bacterial leaking during the procedure, resulting in variations in the number of CFU inoculated. Despite those limitations, local infections in the urinary tract are an important tool to investigate the contribution of *K. pneumoniae* virulence factors to disease and develop new therapeutics/vaccines to control UTIs.

Transurethral inoculation of *K. pneumoniae* may also be employed to evaluate ascending UTIs—an important feature in urinary tract infections in humans. It is also a simpler technique that requires some level of expertise, but the animal manipulation does not demand specific equipment or local surgery, which includes the recovery process as a potential issue. Therefore, it should be considered as the gold standard technique for UTI studies. Conversely, given the prevalence of catheter-induced UTIs in hospital dwellings, models that mimic this type of device should be considered when evaluating nosocomial infections.

### Non-mammal disease models in *Klebsiella pneumoniae*

Very few models were described for *K. pneumoniae* infections using animals other than rodents. A recent work by Zhang and colleagues have used zebrafish (*Danio rerio*) to investigate the variations in infection by different *K. pneumoniae* strains, and the innate immune responses mounted against these bacteria. The fish were infected by immersion in a bacterial suspension for 8 or 24 h, followed by organ collection and bacterial counting. The intestines were the organs with the highest bacterial loads (Zhang X. et al., 2019).

The zebrafish model is also suitable to analyze neutrophil and macrophage migration during *K. pneumoniae* infection, since they harbor a mammalian-like innate immune system; however, for evaluation of adaptive responses, a mammalian model is required (Zhang X. et al., 2019). Another limitation of this model is the lack of control in the bacterial loads infecting each animal since they are immersed in the suspension. In the same work, the authors used a *Galleria mellonella* infection model. This is a convenient model to determine the virulence of a bacterial strain or even to compare two or more strains’ behavior during infection, which was the case in the cited study. The authors were able to screen among different strains and select the one with the highest potential of causing infection after colonization, which was achieved via injections with 1×10^6^ CFU, in 10 μL, of a *K. pneumoniae* suspension, using a Hamilton syringe into the haemocoel of the larvae proleg (Zhang X. et al., 2019). The signs of a successful infection included changes in pigmentation, from the typical clear color to a darker tone, combined with the lack of motility are signs of lethality in the larvae (Harding et al., 2013).

Another *in vivo*, non-vertebrate model platform, used to evaluate therapies against *K. pneumoniae* infections is the nematode *Caenorhabditis elegans*. In a phage therapy assay, *K. pneumoniae*, *E. coli* and *Enterobacter cloacae* strains were used in a liquid-phase infection model, in which, either the prophylactic or therapeutic use of the bacteriophages were able to increase the survival of *C. elegans* against the pathogens alone or in combination (Manohar et al., 2022). Antimicrobial screening studies were also conducted in *C. elegans*; using different concentrations of multiple antimicrobials in a lethality assay against carbapenem-resistant *K. pneumoniae* isolates. The lethality model showed comparable results to the usual Kirby-Bauer disk diffusion resistance test, for most of the drugs. Employing the same survival model, dose-dependent drug toxicity was also analyzed and provided data consistent with preconized protocols. Based on the screening results, a therapy protocol was selected and used in two hospitalized patients (Yao et al., 2022).

Comparative virulence of *K. pneumoniae* isolates was assessed in a *Drosophila melanogaster* (fruit fly) model. Multirresistant isolates with matte and mucoid phenotypes were tested in colonization/ lethality assay. The animals (3–5 days old) were injected with increasing bacterial loads using a 10-μm needle. The isolates only killed a small number of flies, however, the CFU counts in the mucoid phenotype group was higher when compared to the non-mucoid strains. It is unclear if the lethality was not achieved due to the type of bacterial strain, or the fly model is not adequate for this type of analysis (Lee et al., 2018).

Overall, animal models of *K. pneumoniae* employing non-mammalian species are still scarce, and future studies are required to assess the potential applicability of the data to human infections. Nevertheless, the current results demonstrate that these models could contribute to our understanding of *K. pneumoniae* pathogenesis, more specifically in drug screening or comparative virulence assays.

## Limitations and shortcomings of animal models for the study of *Klebsiella pneumoniae* infections

As described throughout this review, mice studies represent the most used model to investigate *K. pneumoniae* infections in different mucosal sites. However, this model has important limitations such as a high intrinsic resistance to *K. pneumoniae* strains, including clinical strains, and differences in *K. pneumoniae* lethality in different mice lineages (Mizgerd and Skerrett, 2008). Meanwhile, primates appear to be more susceptible to *K. pneumoniae* infections, which makes sense when considering the high anatomical and immunological similarities between humans and primates. However, they are extremely expensive and require specialized animal facilities, which greatly limits their use. There is also an ethical concern regarding the use of highly sentient beings in experimentation when other options are available. Therefore, improving the rodent models and combining *in vitro* techniques will provide more applicable results.

A particularity of *K. pneumoniae* is the differences between the classical and hypervirulent strains. Often, hypervirulent strains are lethal in mouse studies, however, that may not be the intention of the study (colonization studies, for example). There is a lack of well described bacterial strains leading to non-lethal infections. Unlike infection models for better stablished pathogens, such as *Streptococcus pneumoniae* (Chiavolini et al., 2008), the choice of the bacterial strains ends up being difficult, since there are few correlates of virulence or lethality in mice. Recent studies investigating the genetic diversity of *K. pneumoniae* clinical isolates (Martin et al., 2023) and correlation of certain virulence factors with disease potential in mice (Russo et al., 2021) provide valuable insights in the pathogenesis of *K. pneumoniae*. However, the data are from systemic infections resulting from subcutaneous or intraperitoneal challenges; it remains to be investigated whether the same is true for mucosal infections. Even when looking at survival as an endpoint, the current animal models may not represent the natural stages of disease development seen in patients infected with *K. pneumoniae,* which limit the extrapolation of the results to clinical scenarios.

As an option to replace the use of animals in the basic research of UTI treatments, an *in vitro* model was developed, using real urine, simulating the physiological conditions of the urinary tract with a Foley catheter passing through the cap of a conical tube, surrounded by tryptone soy agar. The bacterial growth was evaluated by analyzing the bacterial counts in the catheter (Gaonkar et al., 2003).

Techniques for animal replacement involve the use of artificial environments that mimic human niches, as in 3D cell culture or microfluidic systems, exemplified by lab- or organ-on-a-chip technologies. The tridimensional cell culture models resemble natural tissue structures, resulting in functional organoids maintained in culture conditions, allowing an increase in the development speed in the pre-clinical stages of research. Though the technique is in constant evolution, there are still limitations and questions about its reproducibility and biological significance (Shah et al., 2023). Similarly, the microfluid chip models are also a promising alternative, being able to reproduce physiological events such as the physical forces and cell organization (Feaugas and Sauvonnet, 2021). However, there are also drawbacks in its application: the systems are still in validation process, without well-established standard procedures, and the complexity of the human systems make it almost impossible to replicate identically (Leung et al., 2022). Therefore, although animal replacement techniques have important limitations, especially considering systemic studies (like for instance, in vaccine development), there is definitely a huge potential for development of novel therapies.

Another option is the substitution of the typical vertebrate models by invertebrates, such as *C. elegans*, *D. melanogaster*, or *Galleria mellonella*. Just like any *in vivo* model, there are advantages and disadvantages. Invertebrates are cheaper, often easy to manipulate and are less complex when compared to vertebrate mammals. Nonetheless, there are also limitations in the application of those models. In the *G. mellonella* case in particular, the larvae has an innate immune system somewhat similar to ours, but it lacks an adaptative response (Ménard et al., 2021). In *K. pneumoniae* infection models, *G. mellonella* has been used with vastly different goals, such as to evaluate phage-mediated survival (Feng et al., 2023), synergistic activity of antimicrobials (Ribeiro et al., 2023) and pathogenicity (Liu et al., 2023). However, when used to compare hypervirulent and common *K. pneumoniae* strains, the model did not reproduce the same differences observed in outbred mice (Russo and MacDonald, 2020).

Similarly, *C. elegans* has been used as a platform to study *K. pneumoniae* infection, antimicrobial drugs activity (Yao et al., 2022) and bacteriophage therapy efficacy (Manohar et al., 2022). Survival studies and analysis of *K. pneumoniae* virulence can also be achieved using *D. melanogaster* (Lee et al., 2018). There is a description of colonization and bacterial metastasis in zebrafish, using different clinical *K. pneumoniae* strains isolated from patients’ gut microbiota (Zhang X. et al., 2019). All those studies represent interesting approaches to investigate some traits of *K. pneumoniae* infections, but they do not allow a detailed analysis of the host immune responses during infection, nor the mechanisms responsible for increased survival after vaccination/treatment. A recent review in vaccine development against *K. pneumoniae* can be found in Assoni et al. (2021).

## Conclusion

Mammals, and specifically, rodents like mice and rats, are the most widely models for studying *K. pneumoniae* infection. The anatomical similarities and comparable immune responses, combined with the easy handling and availability of a variety of strains make these animals the gold standard in *K. pneumoniae* research *in vivo*. However, they present important limitations, instigating the development of more physiological approaches, which mimic the hallmarks of human infections and allow a better understanding of the host-pathogen interactions during disease. Transgenic animals with humanized immune systems, and/or those with humanized microbiota may represent a more physiological platform to study *K. pneumoniae* infections. Additionally, inoculation procedures that closely emulate the natural pathogenic process may provide a better understanding of the different infection stages.

The choice of the most appropriate animal model must also consider the anatomical and immunological specificities of a particular strain, which greatly impact the outcome of the study. The use of neutropenic mice, for instance, usually provides increased bacterial burden, while anatomical differences in the urinary tract may favor more severe diseases. These traits, as well as the use of animals with specific mutations, could provide reproducible and stable infections, allowing the evaluation of intended outcomes, which could be further correlated with human patients.

Given the ability of *K. pneumoniae* to colonize different host niches, causing diseases of variable severity, it is important to develop models that replicate the conditions (from the pathogen and the host) associated with bacterial persistence in each host tissue. In that sense, whole transcriptome and proteome profiling may help identify the virulence factors involved in different stages of disease and how these factors interact to promote bacterial persistence in different sites within the host. Furthermore, the use microbial communities (co-infection) may better represent the natural environment in which *K. pneumoniae* colonizes the host, since different microbes are known to cooperate or compete for the same host niche. Finally, since *K. pneumoniae* is known to form biofilms *in vitro* and *in vivo*, the use of biofilm bacteria instead of planktonic may help unveil disease mechanisms which are unique to this bacterial phenotype. This has already been demonstrated during colonization of the bladder and may also be important in other tissues such as the lungs.

## Author contributions

LA: Writing – review & editing, Writing – original draft. AC: Writing – original draft. BV: Writing – original draft. BM: Writing – original draft. AL: Writing – original draft. TC: Writing – review & editing, Supervision, Conceptualization. MD: Writing – review & editing, Writing – original draft, Supervision, Funding acquisition, Conceptualization.
